# Prehospital administered fascia iliaca compartment block by emergency medical service nurses, a feasibility study

**DOI:** 10.1186/1757-7241-22-38

**Published:** 2014-06-23

**Authors:** Els Dochez, Geert J van Geffen, Jörgen Bruhn, Nico Hoogerwerf, Harm van de Pas, Gertjan Scheffer

**Affiliations:** 1 Ambulanceservice, South East Brabant Region, Eindhoven, The Netherlands; 2 Dept of Anesthesiology, Pain and Palliative care, Radboud University Medical Centre, PO Box 9101, 6500 HB Nijmegen, The Netherlands; 3 HEMS Lifeliner 3, Radboud University Medical Centre, Nijmegen, The Netherlands; 4 Acute Care Network, Brabant, Tilburg, The Netherlands

**Keywords:** Regional anesthesia, Fascia iliaca compartment block, Proximal femur fracture, EMS-nurses

## Abstract

**Introduction:**

Patients with a proximal femur fracture are often difficult to evacuate from the accident scene. Prehospital pain management for this vulnerable group of patients may be challenging. Multiple co-morbidities, polypharmacy and increased age may limit the choice of suitable analgesics. The fascia iliaca compartment (FIC) block may be an alternative to intravenous analgesics. However this peripheral nerve block is mainly applied by physicians.

In the Netherlands, prehospital emergency care is mostly provided by EMS-nurses. Therefore we examined whether well-trained EMS-nurses are able to successfully perform a FIC block in order to ensure timely and appropriate effective analgesia.

The study was study was registered in the Netherlands Trial Register (NTR-nr 3824).

**Methods:**

Ten EMS nurses were educated in the performance of a FIC-block. Indications, technique, side-effects and complications were discussed. Hereafter the trained EMS-nurses staffed ambulance teams were dispatched to patients with a suspicion for a proximal femur fracture. After confirmation of the diagnosis, the block was performed and 0.3 ml/kg lidocaine (10 mg/ml) with adrenaline 5 μg/ml was injected. The quality of pain relief, occurrence of complications and patient satisfaction were evaluated.

**Results:**

In 108 patients a block was performed. One hundred patients could be included. Every EMS nurse performed at least 10 FIC blocks. The block was effective in 96 patients. The initial median (NRS)-pain score decreased after block performance to a score of 6 (after 10 minutes), 4 (after 20 minutes) and 3 (after 30 minutes). At arrival at the Emergency Department the median pain score was 3. Dynamic NRS-pain scores when transferring the patient from the accident scene to the ambulance stretcher, during transportation to the hospital and when transferring the patient to a hospital bed were, 4, 3 and 3.5 respectively. Patient satisfaction was very high. No complications were noted.

**Conclusion:**

Additional educated EMS-nurses are able to successfully perform a FIC-block for providing acute pain relief to patients with a suspected proximal femur fracture.

## Introduction

Emergency medical services (EMS) nurses providing emergency care are frequently confronted with patients suspected with proximal femur fractures (PFF). In people older than 70 years the incidence is approximately 0.5% and due to the ageing population a rise in hospital admissions for suspected proximal femur fractures will be expected [1].

After a fall, these elderly people with PFF’s may be difficult to evacuate and often multiple painful transfers are necessary (from the floor to the ambulance stretcher, hospital and radiology bed) before a definite diagnosis can be made. Prehospital pain management for this vulnerable group may be challenging. Multiple co-morbidities and polypharmacy may limit the choice of suitable analgesics. The cognitive impairment, either as a result of dementia or acute confusion as a result of the injury may contribute to inadequate administration of analgesia of analgesic drug [2]. Almost half of cognitively intact individuals with a PFF report severe to very severe pain preoperatively. Although physicians prescribe opioids to treat pain, patients older than 65 years hospitalized for these fractures receive only less than 25% of opioid analgesics prescribed [3].

The administration of a fascia iliaca compartment (FIC) or femoral nerve block is a safe alternative and has demonstrable benefits to intravenous analgesics [4].

A number of studies support the use of femoral nerve or fascia iliaca compartment block for pain relief in the emergency department after femur fractures [5-7]. This peripheral block has become a part of an integrated care pathway, sometimes performed by advanced nurse practioners who have the decision making skills and clinical competencies for expanded practice beyond that of a EMS-nurse [8,9].

Also in prehospital emergency care this nerve block has been successfully applied by physician staffed emergency teams [10-12].

In the Netherlands prehospital emergency care is mostly provided by EMS-nurses. Therefore we examined whether well-trained EMS-nurses are able to successfully perform a fascia iliaca compartment block, in order to ensure timely and appropriate effective analgesia, as evaluated by a decrease in NRS-score with a least two points, to all patients with a suspected proximal femur fracture.

## Patients and methods

Ten EMS-nurses were trained in the performance of a FIC-block. A theoretical training in anatomy, pharmacology of local anesthetics, recognition and treatment of complications and the technical performance of the block was provided. Hereafter the performance of the block was exercised on human cadavers.

At the end of the training a theoretical exam was taken. Then the nurses had to demonstrate and simulate the performance of the FIC-block on a volunteer. The theoretical needle insertion site was verified by ultrasonography.

After successful passing these tests the nurses were considered to be proficient and were authorized by the medical director of the ambulance service to perform the FIC-blocks in order to allocate the right EMS-nurse staffed emergency team to a patients with a suspected proximal femur fracture and to avoid treatment delays, the regional emergency dispatch centre Brabant Zuid-Oost in Eindhoven was also educated in the inclusion and exclusion criteria for patients participating in this study.

Inclusion criteria consisted of age older than 18 years, suspicion of a non-complicated proximal femur fracture, and an unacceptable Numeric Rating Scale (NRS) pain score of four or more. (The NRS pain scale is a numeric scale in which a patient selects a whole number that reflects the intensity of pain, with 0 representing one pain extreme “no-pain” and 10 representing the other pain extreme “the worst pain imaginable”).

Exclusion criteria were decreased conscious level, disorientation for time place and person, hemodynamic instability which was defined as a heart rate lower than 60 or higher than 100 and a systolic blood pressure lower than 90 mmHg, neurovascular damage symptoms, signs of infection at the needle insertion site, impossibility to palpate the femoral artery, Body Mass Index (BMI) higher than 30 kg/m^2^, severe distracting injuries, or allergy for local anesthetics.

After arrival at the scene, the EMS-nurses took a short history. If shortening and exorotation of the affected leg and severe pain in the inguinal or hip region were present on physical examination; then a proximal femur fracture was suspected and a pain (NRS) score was determined and noted.

Intravenous access was obtained and standard monitoring (electrocardiogram, pulse oximetry, noninvasive blood pressure measurement) was applied.

**Figure 1:**
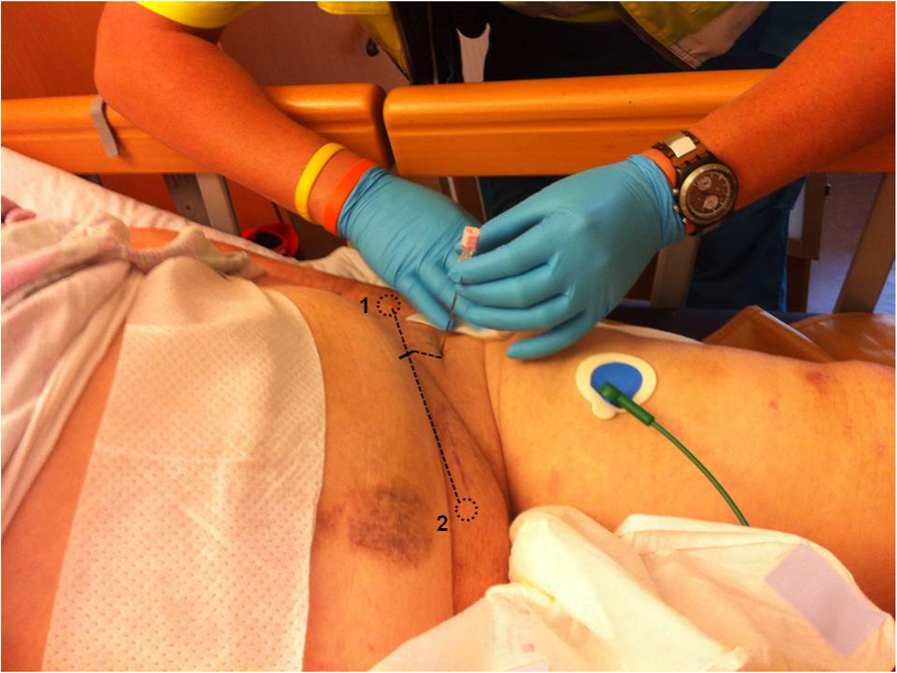
**External reference points on a patient.** The superior iliac spine (1) and pubic tubercle (2) were palpated. The union of the lateral third and medial two-thirds of the inguinal ligament was marked. The needle insertion site is 3 cm below the union.

The procedure was explained to the patient. The single injection FIC block was performed as described by Dalens et al. [4]. The pubic tubercle and the superior iliac spina were palpated. The union of the lateral third and medial two-thirds of the inguinal ligament was marked. The needle insertion site is three cm below the union. For safety reasons the femoral artery was palpated and marked. The puncture site had to be at least one to two cm lateral to the femoral artery (Figure 1).

**Figure 2:**
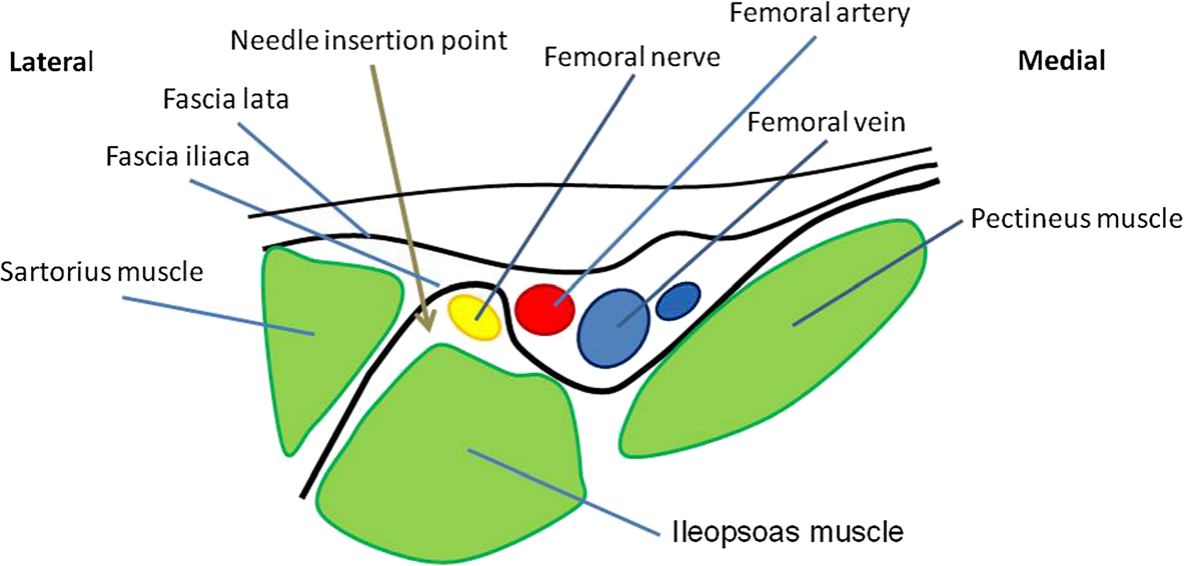
**After skin penetration, two clearly identifiable losses of resistances (clicks or plops) are felt while crossing the fascia lata and fascia iliaca.** After negative aspiration for blood the local anesthetic is injected and will spread below the fascia iliaca and anesthetize the femoral nerve.

From a prefilled flacon 0.3 ml/kg lidocaine (10 mg/ml) with adrenaline 5 μg/ml was drawn up in 10 ml syringes.The skin was cleaned with chorhexidine and under a 70° angle to the skin, the 18-G Tuohy needle (BBraun, Melsungen, Germany) was inserted. Two clearly identifiable loss of resistances (clicks or plops) had to be felt while crossing the fascia lata and fascia iliaca. After negative aspiration for blood the local anesthetic was slowly (over 2 mins) injected after repeated aspiration (Figure 2).

If blood was aspirated then the needle was withdrawn, the reference points checked, and the procedure repeated 0.5 cm more lateral than the initial needle insertion site.

The patient was observed for any signs of local anesthetic systemic toxicity (LAST) such as tingling around the mouth, light headedness, visual disturbances, seizures or arrhythmias. If one of these symptoms occurred, the injection was stopped and oxygen administered. Pain treatment was provided by iv analgesics. Signs of LAST were noted as a complication.

The following data were collected; demographic data, weight of the patient, the ease of the block procedure (1 = very easy, 10 = extremely difficult).

NRS pain scores at arrival at the scene and after 10, 20 and 30 minutes after the performance of the block and at arrival at the Emergency Department of the receiving hospital were obtained.

Also dynamic pain scores (pain scores during manipulation of movement) were noted after movement to the ambulance stretcher, during ambulance transporting and when transferring from ambulance stretcher to the hospital bed.

In the cutaneous distribution of the femoral nerve, sensibility was tested at the medial mid-femoral and medial site of the lower limb, using cold alcohol swabs.

A FIC-block was considered successful if the NRS-pain score decreased with at least two points.

If 30 minutes after block performance the patient considered the pain as unacceptable and the NRS pain score had not decreased to three or lower, than the (nationwide) Dutch Ambulance protocol (LPA 7.2) was followed and fentanyl 2 μg/kg was injected intravenously. If needed every three minutes an additional dose of 0.5 μg/kg fentanyl was given until acceptable pain scores or NRS score lower than four was reached. The need for additional analgesics was noted.

In order to obtain informed consent all patients were visited during their hospital stay. During this visit they were also asked about their satisfaction with the provided pain relief before admission in the hospital. The patient satisfaction score was noted (1 = absolutely not satisfied, 10 = very satisfied).

Neurological complications were noted after reviewing the medical record.

Descriptive statistics were used to describe and summarize the data obtained in this study. Demographic data are presented as mean (standard deviation). NRS pain scores are presented as median (first quartile – third quartile). Calculations were made with Microsoft Office Excel 2007 (Microsoft, Redmond, USA).

The Medical Ethics Committee of Arnhem-Nijmegen approved a waiver of informed consent for performing the FIC block but written informed consent was obtained for the use of the documented data. The study was registered in the Netherlands Trial Register which is equivalent to the ClinicalTrials.gov registry (NTR-nr 3824). Progress of the study and safety of the patients was reviewed by an independent Data Safety Monitoring Board.

## Results

Patients were included between November 1, 2012 and December 1, 2013. The regional emergency dispatch centre allocated 110 times the trained EMS-nurses to a patient with a suspected proximal femur fracture. At arrival two times no fracture was suspected. In 108 patients a fascia iliaca compartment block was performed. However six patients were not able to give informed consent due to postoperative confusion and two due to severe postoperative complications. So 100 FIC-block procedures could be included in this study.

**Figure 3:**
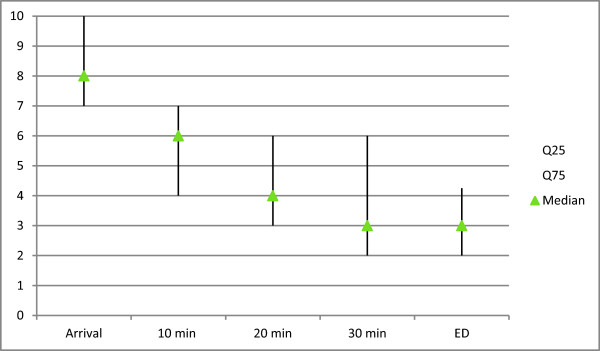
Median first and third quartile NRS pain scores at arrival, 10, 20, 30 minutes after FIC block performance and at arrival at the emergency department (ED) (NRS score, 0 = absolute no pain, 10 = most extreme pain).

**Figure 4:**
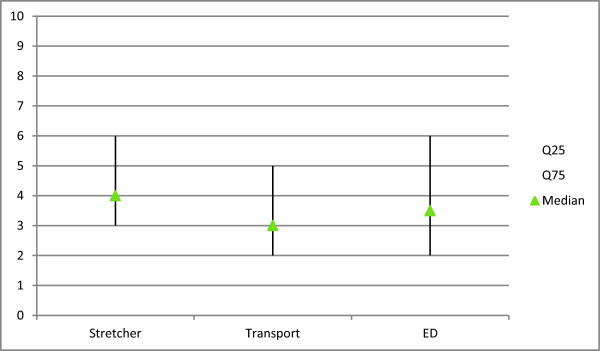
**Median (dynamic) NRS pain scores, at transfer to ambulance stretcher, ambulance transport to the hospital and at transfer from stretcher to hospital bed.** (NRS score, 0 = absolute no pain, 10 = most extreme pain).

In 80 women and 20 men a block was performed. The mean age of the patients was 81 years. (SD = 9.4) and the mean BMI was 24 (SD = 3.8).Median (at rest) NRS pain scores at arrival decreased, 10, 20 and 30 minutes after FIC-block performance (Figure 3). Median (dynamic) NRS pain scores at transfer to ambulance stretcher, during ambulance transport and transfer from stretcher to hospital bed are shown in Figure 4. (NRS score, 0 = absolute no pain, 10 = most extreme pain).

In four patients the NRS-pain score did not decrease with a least two points. So the block was considered successful in 96% of the patients. The median decrease in pain score between arrival at the scene and arrival in the ED was 5 (min 0, max 5). At arrival in the ED, 75% had a NRS-pain score of four or lower. Loss of sensibility in the femoral nerve distribution was detected in 88% of the patients. Patients were satisfied with the provided pain relief and the median satisfaction score was nine. No complications were observed. Seven patients complained about nausea during transportation.

In 93 patients the suspected diagnosis of proximal femur fracture PFF was confirmed after physical and X-ray examination by a physician.

The other seven patients were diagnosed with contusion of the hip and the FIC block was successful in six of them.

## Discussion

This study demonstrates that trained nurses can succesfully perform a FIC block in patients with a suspected proximal femur fracture. The block was successful in 96%. The relative high successrate may be the result of our definition of block success, but if we would have defined block success as a decrease in NRS-pain score with more than three points the success was still 90%. At arrival in the ED, 75% of the patients had a pain score of four or lower. This is slightly better than previously reported results (70%) of a femoral nerve block performed by nurses in the ED [7,8]. In another study, non-anesthesiologists performed in 94% of the patients a succesful FIC-block prehospitally, while block success was defined as loss of sensibility in the femoral nerve territory. This is slightly higher than our result (88%) [12].

Regional anesthestic procedures are significantly more difficult to learn than manual skills necessary for providing general anesthesia. Learning manual skills depends on many variables. Besides differences in individual sensorimotor skills of the operator, the exposure to a sufficient number of cases influences the learning skills [13]. Our inexperienced nurses obtained a relative high successrate, which might be explained by the low complexity of this block and the effectiveness of the training. In order to obtain and maintain competency in the performance of the FIC-block, it is necessary that the trained nurses are able to regularly practice their skills. Therefore we believe that this regional block technique should be exclusively applied by a selective group of additionally trained nurses.

For providing acute pain relief to trauma patients, the use of peripheral nerve blocks has advantages over the present technique of administering opioids, or midazolam and ketamine. The risk of respiratory complications, sedation, nausea and vomiting is lower. Moreover nerve blocks may provide longer lasting analgesia [14,15]. However it should be noted that administering peripheral nerve blocks for providing additional pain relief after inadequate intravenous analgesia with opioids bares the risk of inducing respiratory depression [16].

The FIC-block is a peripheral nerve block which was described by Dalens in 1989 [4]. It has a fast onset and provides effective pain relief [6-12,17].

The FIC block based on external anatomical landmarks is an easy and relative safe block. Besides a needle, syringe and local anesthetic no other equipment is necessary. The needle insertion site is located in some distance from the femoral artery and nerve which minimizes the risk on intravascular injection nd neurological injury. Complications as nerve damage and intravascular injection are seldom described [4].

Recently ultrasonography has also been used for the performance of this nerve block [18,19]. However, because of the high costs for the ultrasound equipment, it is expected that this technique will not be widely applied in the prehospital setting, although the use of ultrasound certainly has benefits over the blind technique.

Compared to other local anesthetics, lidocaine is one of the safest local anesthetics. It was injected in a dose of 3.0 mg/kg, this is only half of the recommended maximal dose described (6 mg/kg) [20]. Lidocaine is a short-acting local anesthetic with a duration of action of 2 to 4 hours. This implies that for a more long acting pain relief the block needs to be repeated with a longer acting local anesthetic, such as ropivacaine or L-bupivacaine. However these local anesthetics are less safe and therefore less suited for prehospital administration by non-anesthesiologists.

An advantage of administering short acting local anesthetic for pain relief is that the block quickly wears off and this allows early mobilisation in case no surgical intervention is necessary.

In patients with a proximal femur fracture, hip flexion is limited to 15 degrees. However after a succesful FIC-block due to analgesia and relaxation of the quadricpes muscle, hip flexion increases to 53 degrees [21]. This allows that a patient from supine position may be mobilised to a half sitting position. For EMS nurses this is a great advantage when a patient after a fall and lying in supine position, needs to be evacuated from a difficult to accessible accident scene to a ambulance stretcher. A FIC-block allows a more easy and less painful evacuation. In the hospital, after confirmation of the diagnosis it allows more easy positioning for spinal anesthesia for surgical intervention [22].

A limitation of this study is that the effectiveness of the block is not tested by an independent observer. However for practical and logistical reasons it was not possible to add 10 observers to 10 solely working ambulance teams. Moreover the study is not powered to draw any conclusions on the incidence of complications which may occur during or after the performance of a FIC-block by paramedics.

In conclusion this study demonstrates that educated EMS-nurses can effectively perform FIC-blocks for acute pain relief in patients with a suspected proximal femur fracture. Further research may reveal whether this method of pain relief may lead to less complications than the present methods for providing acute pain relief.

## Competing interests

The authors declare that they have no competing interests.

## Authors’ contributions

ED contributed to the design of the study, educated the EMS-nurses and was responsible for acquisition of data and monitoring the study. GJvG designed the study, educated the EMS-nurses, reviewed and interpreted the data and drafted the manuscript. JB and NH contributed to the design of the study, reviewed and interpreted the data and contributed in writing the manuscript. HP and GJS reviewed and interpreted the data and revised the manuscript. All authors have given final approval of this manuscript for publishing and agree to be accountable for all aspects of this work.
